# Supplementary material for: Content-rich biological network constructed by mining PubMed abstracts
Source: BMC Bioinformatics. 2004 Oct 8;5:147. doi: 10.1186/1471-2105-5-147 (PMC528731; doi:10.1186/1471-2105-5-147)
Supplement: Additional File 2 — The original results of the above study (non-essential files are deleted to keep the file size under the limit set by BMC bioinformatics). [file 1471-2105-5-147-S2.bz2 › chilibotAdditionalFile2/dip05/713ID8175697E18050/html/SECA_SECB.html]

 


 **SECA** and **SECB** 
  
Found 76 abstracts in PubMed, retrieved 05.  
 

 What does Google say? 
 PDF only 
| .edu only 

---

**Interactive relationship** (e.g. stimulation, inhibition, etc)

**Neutral relationship**- In this latter role  **SecB**  demonstrates specific binding to a unique partner,  **SecA** .  Ref: 12475171 Cell Mol Life Sci, 2002

**Non-interactive relationship** (e.g. studied together, co-existance, homology, etc.)

- Export after  **SecA**  depletion was observed in cells that lack  **SecB** , the primary targeting factor for OMPs, but was abolished by partial inactivation of DnaK.  Ref: 12403776 J Biol Chem, 2002
- The characterization of a spontaneous TorA signal peptide mutant TorA\*, in which the two arginine residues in the c region had been replaced by one leucine residue, showed that the TorA\* MalE mutant precursor had acquired the ability for efficiently using the  **SecB**   **SecA**  pathway.  Ref: 12700260 J Bacteriol, 2003
- In Escherichia coli, the  **SecB**   **SecA**  branch of the Sec pathway and the twin arginine translocation Tat pathway represent two alternative possibilities for posttranslational translocation of proteins across the cytoplasmic membrane.  Ref: 12700260 J Bacteriol, 2003
- In Escherichia coli, three different types of proteins engage the SecY translocon of the inner bacterial membrane for translocation or insertion 1 polytopic membrane proteins which prior to their insertion into the membrane are targeted to the translocon using the bacterial signal recognition particle SRP and its receptor, 2 secretory proteins which are targeted to and translocated across the SecY translocon in a  **SecA**  and  **SecB**  dependent reaction and 3 membrane proteins with large periplasmic domains, requiring SRP for targeting and  **SecA**  for the translocation of the periplasmic moiety.  Ref: 12682042 J Biol Chem< MedlineTA>J Biol Chem, 2003
